# Elevated phosphorylation and activation of PDK-1/AKT pathway in human breast cancer

**DOI:** 10.1038/sj.bjc.6602862

**Published:** 2005-11-15

**Authors:** H-J Lin, F-C Hsieh, H Song, J Lin

**Affiliations:** 1 Division of Medical Technology, School of Allied Medical Professions, College of Medicine and Public Health, The Ohio State University, Suite 535A, Atwell Hall, 453 West 10th Street, Columbus, OH 43210, USA; 2 Center for Childhood Cancer, Columbus Children's Research Institute, Columbus, OH 43210, USA; 3 University of Michigan Comprehensive Cancer Center, Ann Arbor, MI 48109, USA; 4 Integrated Biomedical Science Graduate Program, College of Medicine and Public Health, The Ohio State University, Columbus, OH 43210, USA; 5 Ohio State Biochemistry Graduate Program, College of Medicine and Public Health, The Ohio State University, Columbus, OH 43210, USA; 6 Ohio State University Comprehensive Cancer Center, College of Medicine and Public Health, The Ohio State University, Columbus, OH 43210, USA; 7 Department of Pediatrics, College of Medicine and Public Health, The Ohio State University, Columbus, OH 43210, USA

**Keywords:** PDK-1, AKT, mTOR, p70S6K, phosphorylation, breast cancer

## Abstract

Activation of kinases signalling pathways contributes to various malignant phenotypes in human cancers, including breast tumour. To examine the possible activation of these signalling molecules, we examined the phosphorylation status in 12 protein kinases and transcription factors in normal primary human mammary epithelial cells, telomerase-immortalised human breast epithelial cell line, and two breast cancer lines, MDA-MB-468 and MCF-7, using Kinexus phosphorylated protein screening assays. The phosphorylation of FAK, mTOR, p70S6K, and PDK-1 were elevated in both breast cancer cell lines, whereas the phosphorylation of AKT, EGFR, ErbB2/Her2, PDGFR, Shc, and Stat3 were elevated in only one breast cancer line compared to normal primary mammary epithelial cells and telomerase-immortalised breast epithelial cells. The same findings were confirmed by Western blotting and by kinase assays. We further substantiated the phosphorylation status of these molecules in tissue microarray slides containing 89 invasive breast cancer tissues as well as six normal mammary tissues with immunohistochemistry staining using phospho-specific antibodies. Consistent findings were obtained as greater than 70% of invasive breast carcinomas expressed moderate to high levels of phosphorylated PDK-1, AKT, p70S6K, and EGFR. In sharp contrast, phosphorylation of the same proteins was nearly undetectable or was at low levels in normal mammary tissues under the same assay. Elevated phosphorylation of PDK-1, AKT, mTOR, p70S6K, S6, EGFR, and Stat3 were highly associated with invasive breast tumours (*P*<0.05). Taken together, our results suggest that activation of these kinase pathways by phosphorylation may in part account for molecular pathogenesis of human breast carcinoma. Particularly, moderate to high level of PDK-1 phosphorylation was found in 86% of high-grade metastasised breast tumours. This is the first report demonstrating phosphorylation of PDK-1 is frequently elevated in breast cancer with concomitantly increased phosphorylation of downstream kinases, including AKT, mTOR, p70S6K, S6, and Stat3. This finding thus suggested PDK-1 may promote oncogenesis in part through the activation of AKT and p70S6K and rationalised that PDK-1 as well as downstream components of PDK-1 signalling pathway may be promising therapeutic targets to treat breast cancer.

Breast cancer remains to be a major cause of death in women worldwide, despite improvements in cancer prevention, early detection, treatment, and understanding the molecular pathogenesis. It hence demands an urgent need to unveil the exact mechanism of oncogenesis and tumour progression, which may prelude the development of new strategies for treatment. Breast carcinoma emerges through multistep processes progressing from hyperplasia to premalignant change, *in situ* carcinoma, invasion, and distal metastasis, in concomitant with gain of oncogenic activities and loss of tumour suppressor gene functions.

Protein kinases have been implicated in playing crucial roles in regulating cell growth, metabolic responses, cell proliferation, migration, and apoptosis, which altogether attribute tumorigenesis. Constitutive activation of these protein kinases, mainly by phosphorylation, was implicated in contributing to malignant phenotypes in a number of human cancers including breast carcinoma (Pandolfi, 2004; Saal *et al*, 2005; Zhang *et al*, 2005). These protein kinases are normally phosphorylated by either upstream protein kinases or by autophosphorylation in response to cell signals.

Among growth factors, insulin was well studied in the past few decades. Binding of insulin to receptors initiates phosphoinositol 3-OH-kinase (PI3-K) mediated phosphorylation and hence generates phosphatidyl-inositol 3,4-biphosphate and phosphatidyl-inositol 3,4,5-triphosphate. These lipid products serve as membrane docking sites for recruiting pleckstrin homology (PH)-containing proteins. Among them, 3-phosphoinositide-dependent protein kinase-1 (PDK-1) was identified to be an immediate downstream mediator of PI3-K and was activated upon phosphorylation on residue Ser-241 (Casamayor *et al*, 1999). Activated PDK-1 controls cell proliferation, survival, nutrients uptake and storage, by further activating its downstream AGC (cAMP-dependent, cGMP-dependent, protein kinase C) family of protein kinases including AKT (Alessi *et al*, 1997; Wick *et al*, 2000b), protein-kinase C (PKC) (Le Good *et al*, 1998; Balendran *et al*, 2000), serum- and glucocorticoid-induced kinase (SGK) (Mora *et al*, 2004), and ribosomal p70S6 kinase (Pullen *et al*, 1998). Hence, PDK-1 functions as a pivotal signalling molecule at the apex of complex networks of intracellular signalling (Toker and Newton, 2000; Storz and Toker, 2002).

Among downstream kinases, AKT is one of the most extensively studied PDK-1 substrates. Its activation has been implicated in a number of human cancers by promoting proliferation and inhibiting apoptosis caused by various death stimuli (Del Peso *et al*, 1997; Franke *et al*, 1997; Kulik *et al*, 1997). AKT is activated in response to survival signals from growth factors or cytokines via mechanisms involving PI3-K and PDK-1 (Alessi *et al*, 1997; Franke *et al*, 1997; Kulik *et al*, 1997; Bellacosa *et al*, 1998). Upregulation of AKT by mutation of *PTEN* (a tumour suppressor gene that antagonises AKT pathway) or amplification *PIK3CA* (encodes catalytic subunit of PI3-K) were reported in a number of human cancers including breast carcinoma (Vivanco and Sawyers, 2002; Lee *et al*, 2005; Levine *et al*, 2005). Regarding other receptor tyrosine kinases, breast cancer cells also produce platelet-derived growth factor (PDGF) and its receptors, through autocrine or paracrine mechanisms, to modulate growth, survival, and differentiation of breast tissues (Shao *et al*, 2000).

Another downstream target of AKT is the mammalian target of rapamycin (mTOR) (Scott *et al*, 1998; Nave *et al*, 1999; Sekulic *et al*, 2000). It is a serine/threonine kinase, which phosphorylates at least three downstream substrates: the translation repressor eIF4E-binding protein (4E-BP) (von Manteuffel *et al*, 1997; Gingras *et al*, 1999; Nojima *et al*, 2003); p70S6K, which phosphorylates ribosome protein S6 (Nojima *et al*, 2003); and signal transducer and activator of transcription 3 (Stat3) (Yokogami *et al*, 2000; Kusaba *et al*, 2004). Normally, activation of Stat3 takes place upon cytokine or growth factor stimulation. First, Stat3 was recruited to cytoplasm followed by phosphorylatation on Y705 by receptor-associated kinases. Then activated Stat3 leads to dimer formation, nuclear localisation, and regulation of gene expression (Darnell *et al*, 1994; Zhong *et al*, 1994). Additional phosphorylation on S727 by upstream kinases may augment its transcriptional activation on target genes (Lim and Cao, 2001; Fu *et al*, 2004). mTOR is one of the key kinases that appears to mediate phosphorylation of Stat3 on S727 (Yokogami *et al*, 2000; Yonezawa *et al*, 2004). Taken together, these upstream signalling molecules including PI3-K, PDK-1, AKT and mTOR comprise regulatory pathways controlling cell growth, metabolism, proliferation, differentiation, motility, and survival, which attribute the overall malignancy phenotypes in human cancers (Castedo *et al*, 2002; Carraway and Hidalgo, 2004; Chan, 2004; Xu *et al*, 2004; Zhou *et al*, 2004).

Additional signalling mediators were also implicated in the pathogenesis of breast cancers. c-Jun, a major component of the AP-1 transcription factor, was reported to be either proapoptotic (Cuadrado *et al*, 2004; Qi *et al*, 2004) or antiapoptotic (Johnsto *et al*, 1999; Huang *et al*, 2004) depending upon cellular contexts. One example of cellular regulators involved in the phosphorylation of c-Jun in breast cancer was demonstrated to be the mitogen-activated protein kinase phosphatases (Wang *et al*, 2003). On the other hand, hepatocyte growth factor receptor, c-Met (Edakuni *et al*, 2001; Parr and Jiang, 2001; Elliott *et al*, 2002; Lengyel *et al*, 2005), and the focal adhesion kinase (FAK) regulate multiple cellular processes including growth, differentiation, adhesion, motility and apoptosis. Furthermore, high expression of FAK in breast cancer is related to activation of AKT (Schmitz *et al*, 2005) and to the overexpression of receptor molecules such as ErbB2/Her2 and EGFR (Lemmon, 2003). Lastly, the roles of amplified Shc signalling, substrates for most receptor tyrosine kinases, in breast tumourgenesis and metastasis were studied recently (Mauro *et al*, 1999; Dankort *et al*, 2001; Finlayson *et al*, 2003).

Although increased phosphorylation of AKT and mTOR in breast cancer have been recently reported (Noh *et al*, 2004; Zhou *et al*, 2004; Zhang *et al*, 2005), limited number of studies has contrasted the phosphorylation profiles of large panel of protein kinases among normal and invasive breast cancer tissues. Further, although PDK-1 is one of the crucial downstream targets of PI3-K signalling and a key upstream kinase of AKT, it remains unclear whether PDK-1 is activated in primary breast cancer tissues. Thus, we examined the phosphorylation status of 10 protein kinases as well as two transcription factors (c-Jun and Stat3) in normal primary human mammary epithelial cells (HMEC), telomerase-immortalised human breast cells (TERT), and breast cancer cell lines (MDA-MB-468 and MCF-7) using Kinexus phosphorylated protein screening assays. In addition, consistent results were obtained by using Western blottings and AKT kinase assay in which the phosphorylation levels of PDK-1 were significantly elevated in both human breast cancer cell lines, but at basal level in normal primary HMEC.

Further, we examined whether phosphorylation of PDK-1, mTOR, and p70S6K was similarly elevated in breast primary tumours using tissue microarray slides (TMAs). Interestingly, consistent finding was obtained as increased phosphorylation of these protein kinases was detected in breast cancerous tissues. In sharp contrast, phosphorylation of the same molecules was at negligible levels in normal breast tissues. In addition, the enhanced phosphorylation in kinases was positively associated with disease progression from normal breast epithelial to invasive breast carcinoma (*P*<0.05). Finally, we demonstrated that elevated phosphorylation of PDK-1 was frequently found in high-grade metastasised breast tumours and was significantly associated with increased phosphorylation of its downstream kinases (*P*<0.05), indicating phosphorylation of PDK-1 may activate signalling cascades, which altogether contribute to breast cancer.

## MATERIALS AND METHODS

### Breast cell lines and culture

Normal primary HMEC were purchased from Cambrex Inc. (East Rutherford, NJ, USA) and were maintained in mammary epithelial growth medium as recommended by the manufacturer. Telomerase immortalised breast epithelial (TERT) and the spontaneous immortalised mammary epithelial cells MCF10A, gifts from Dr Stephen Ethier at the Karmanos Cancer Institute, Wayne State University (Detroit, Michigan), were cultured in F-12 medium containing 5% FBS, 5 *μ*g ml^−1^ insulin, 1 *μ*g ml^−1^ hydrocortisone, 10 ng ml^−1^ EGF (Sigma, St Louis, MO, USA), 100 U ml^−1^ penicillin and 100 *μ*g ml^−1^ streptomycin (Invitrogen Life Technologies, Carlsbad, CA, USA). Human breast cancer cell lines, MCF-7 and MDA-MB-468, were purchased from American Type Culture Collection (Manassas, VA, USA) and were maintained in 90% Dulbecco's modified Eagle's medium supplemented with 10% foetal bovine serum (FBS) and antibiotics (100 U ml^−1^ penicillin, 100 *μ*g ml^−1^ streptomycin) (Invitrogen Life Technologies, Carlsbad, CA, USA) at 37°C in 5% CO_2_.

### Examination of the phosphorylation profiles on kinases derived from normal breast and breast cancer lines

Cultured cells at subconfluent healthy state were collected in ice-cold PBS containing 1 mM PMSF, 2 mM sodium orthovanadate (Sigma Co., St Louis, MO, USA), 2 mM NaF, and complete protease inhibitor cocktail (Boehringer Mannheim, Germany) and then were lysed in modified radioimmunoprecipitation (RIPA) buffer containing the above protease and phosphatease inhibitors. Quantitation of total protein concentrations in the cell lysates was determined by Bio-Rad Bradford assay. In total, 100 *μ*g of total proteins derived from HMEC, TERT, MCF-7, or MDA-MB-468 were subjected to protein phosphorylation analysis on 12 cell signalling molecules using Kinexus KCPS-1.0 phosphoprotein profiling screen software (Kinexus Bioinformatics Corporation in Vancouver, British Columbia, Canada). This analysis combines proprietary methodologies with the analytical techniques involving gel electrophoresis, immunoblotting, and protein visualisation via enhanced chemiluminescence (ECL). Briefly, the KCPS-1.0 screen detects the target proteins in two steps. First, molecules were separated by gel electrophoresis based on their molecular weights, and then were detected by their immunoreactivity with highly validated phospho-specific antibodies. The panel of antibodies used in this study harbour specific reactivities with phosphorylated AKT (S473), c-Jun (S73), c-Met (Y1230/Y1234/Y1235), EGFR (Y1068), ErbB2/Her2 (Y1248), FAK (Y576), mTOR (S2448), p70S6K (T389), PDGFR (Y716), PDK-1 (S241), Shc (Y239/Y240), and Stat3 (S727). The resulting immune complexes were subjected to a multiplexing apparatus, and the quantitation of the bands is visualised using ECL followed by a highly sensitive imaging system with a 16-bit camera and a quantitation software to analyse the chemiluminescent samples. Each sample's immunoblot was scanned at its maximum time to ensure that the signal from the strongest immunoreactive protein on the immunoblot remains below saturation. It thus provides accurate quantitation over a 2000-fold range of linearity.

### TMA slides and immunohistochemistry (IHC) staining

Multiple sets of breast TMA slides were purchased from BioChain Institute, Inc. (Hayward, CA, USA), which altogether gave rise to six normal breast epithelium and 89 of invasive breast carcinoma. Among them, 83 of breast tumours were categorised into four stages as described (Elston and Ellis, 1991) and were recommended by the American Cancer Society, whereas the remaining six breast tumours were uncertain regarding disease stages. IHC staining was performed utilizing immunoperoxidase techniques described by the manufacturer. Reactions were carried out in TBS buffer (20 mM Tris plus 140 mM NaCl, pH 7.6) supplemented with 0.1% Tween-20 unless specified. Briefly, slides that contain paraffin sections were deparaffinised in xylene, rinsed through two changes of 100% ethanol, rehydrated in graded ethanol (95–70%) and water, and were further subjected to heat-induced epitope retrieval in either 1 mM ethylenediaminetetraacetic acid (EDTA, pH 8.0, for phosphorylated EGFR and Stat3) or 10 mM citrate buffer (pH 6.0, for the remaining kinases in this study). Endogenous peroxidase activity was blocked with 3% hydrogen peroxide and nonspecific binding was prevented by incubation with 3% normal serum for 1 h at room temperature. Slides were then incubated with adequately diluted phosphor-specific primary antibodies (in TBS containing 3% normal serum) at 4°C for an overnight. After washing with TBS buffer, the primary immune complexes were reacted with biotinylated secondary antibodies provided in ABC kits (Vector Laboratories, Inc., Burlingame, CA, USA) and the resulting complexes were detected upon the addition of a chromogen, 3-amino-9-ethylcarbazole (AEC, DakoCytomation, Inc., Carpinteria, CA, USA). After counterstaining of nuclei with haematoxylin (Fisher Scientific, Middletown, VA, USA), slides were sealed in Crystal/Mount aqueous-based mounting Medium (Biomedia Inc., Texarkana, Arkansas).

The primary antibodies used in this study were purchased from Cell Signaling Technology Inc. (Beverly, MA) and were highly validated by the manufacturer. The following antibodies were applied in IHC staining: antitotal PDK-1 (recognises all forms PDK-1 regardless phosphorylation status,1 : 50 dilution, catalog # 3062) and phosphor-specific antibodies including PDK-1 (S241, 1 : 50 dilution, catalog # 3061); AKT (T308 and S473, 1 : 25 dilution, catalog # 4056 and 9277 respectively); mTOR (S2448, 1 : 50 dilution, catalog # 2971); p70S6K (T389, 1 : 300 dilution, catalog # 9206); S6 ribosomal protein (S235/236, 1 : 50 dilution, catalog # 2211); Stat3 (S727 and Y705, 1 : 50 dilution, catalog # 9134 and 9135, respectively); and EGFR (Y1068, 1 : 50 dilution, catalog # 2234).

The overall intensity of IHC staining on ductal and lobular regions within each tissue was scored by eyes with the aids of an inverted microscope and was graded as following: 0, negative staining; 1, weak staining; 2, moderate staining; and 3, intensive staining. Scores were independently graded by two researchers (HL and JL). In order for a tissue to be grouped as positive for phosphorylation, the score had to be 2 or greater.

### Statistic analysis

Statistic analysis was carried out using SPSS software version 13.0 (SPSS Inc., Chicago, IL, USA).

Student's *t*-test was to analyse if phosphorylated kinases correlate with invasive breast carcinomas whereas the Bivariate Pearson *χ*^2^ tests to relate the phosphorylation of PDK-1 to other kinases that were studied in this report. Correlation was considered to be significant if the correlation coefficient *P* is less than 0.05. All *P*-values were two-sided.

### Western blot analysis

For Western blot analysis, 100 *μ*g of proteins derived from total cell lysates were resolved on SDS–PAGE and transferred onto PVDF membrane. The same phospho-specific antibodies that were used in IHC were used to detect the corresponding phosphorylated proteins on the membrane. Monoclonal antibody to glyceraldehydes-3-phosphate dehydrogenase (GAPDH; Biochemistry Lab, Temecula, CA, USA) was used to verify uniform protein loading. All blots were scanned with the Image Quant software using an electrochemifluorescence (ECF) Western blotting detection system (Amersham Corp., Arlington Heights, IL, USA) on a Molecular Dynamics Storm PhosphorImager (Sunnyvale, CA, USA).

### AKT kinase assay

In total, 500 *μ*g of proteins derived from total cell lysates was immunoprecipitated with immobilised anti-AKT monoclonal antibody (1G1, Cell Signaling Technology, Beverly, MA, USA), and the subsequent AKT kinase assay was performed with GSK-3*α*/*β* as substrates using a commercial kit (Cell Signaling Technology, Beverly, MA, USA). Phospho-specific antibody to GSK-3*α*/*β* (Ser21/Ser9) was used for phosphorylated protein detection.

## RESULTS

### Comparison of protein phosphorylation between normal breast epithelial cells and breast cancer cell lines

Kinase or transcription factor activation by phosphorylation has been implicated to play pivotal roles in human cancers including breast carcinoma. We first compared the phosphorylation profiles of 10 protein kinases and two transcription factors (Stat3 and c-Jun) between normal primary HMEC, TERT, and two breast cancer cell lines, noninvasive MCF-7 and invasive MDA-MB-468 (Krueger *et al*, 2001). We found that phosphorylation of mTOR(S2448) was elevated in about two-fold in both breast cancer cell lines compared to HMEC and TERT. Phosphorylation of PDK-1(S241) was two-fold higher in TERT and was five- to six-fold elevated in both breast cancer cell lines compared to HMEC (Table 1). Further, phosphorylation of p70S6K(T389) was elevated 10- to 35-fold in both breast cancer cell lines while nearly basal level was retained in HMEC and in TERT (Table 1). Phosphorylation of FAK(Y576) was also four-fold elevated in MDA-MB-468 cells and only 1.6-fold elevated in MCF-7 cells (Table 1). On the other hand, the phosphorylation of AKT(S473), EGFR(Y1068), ErbB2/Her2(Y1248), PDGFR(Y716), Shc(Y239), and Stat3(S727) were elevated only in MDA-MB-468 breast cancer cells, but retained basal level in MCF-7 cells compared to HMEC and TERT (Table 1). Phosphorylation of c-Met, however, was nearly unaffected in both breast cancer cell lines (Table 1). In contrast, the phosphorylation of c-Jun was reduced in both breast cancer cell lines and TERT, compared to HMEC (Table 1).

### Elevated phosphorylation of PDK-1(S241) in breast cancer as evidenced by IHC staining on primary tissues fixed on TMA slides and by Western blotting

**Figure 1:**
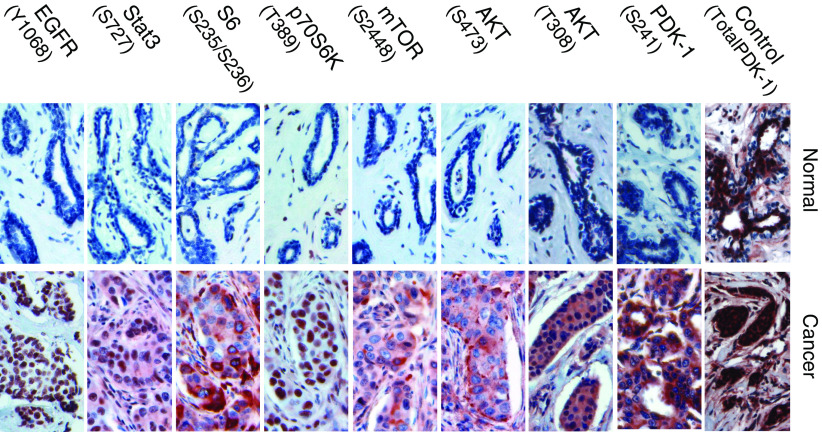
Elevated phosphorylation of PDK-1/AKT pathway in invasive breast cancer. Normal (left panel) and cancerous (right panel) breast tissues fixed on TMA slides were subjected to IHC staining using highly validated phosphor-specific antibodies to examine the phosphorylation status of target proteins. As a control, a primary antibody recognises all forms of PDK-1 were similarly used in IHC staining and the representive example tissues were shown on the top row. The resulting positive immune complexes gave rise to red end product at target antigen sites following the addition of the chromogene, AEC, whereas the unphosphorylated proteins remained colourless. After nuclear staining with haematoxylin (blue dye), representative normal breast tissues (left column) as well as positive examples of breast tumours (right column) were photographed.

To further confirm our investigation on increased phosphorylation in breast cancers, we have applied IHC staining on breast tumours fixed on TMA slides to examine the phosphorylation status of PDK-1, AKT, mTOR, p70S6K, S6, Stat3, and EGFR. Activation of PDK-1 pathway can be mediated by upstream insulin or by other receptor tyrosine kinases and was reported to be critical in regulating survival, metabolism, and cell cycle control (Osaki *et al*, 2004). The roles of PDK-1 in human cancers were implicated by the finding that overexpression of PDK-1 transformed mammary epithelial cells (Xie *et al*, 2003). However, whether the phosphorylation of PDK-1 was elevated in primary breast tumours has not been assessed yet. We therefore examined phosphorylation of PDK-1 at residue S241 in a total of six normal breast tissues and 89 invasive breast carcinomas using IHC staining on TMA slides. Breast cancer tissues, which revealed moderate to high level of PDK-1 phosphorylation (scores 2 and 3), were grouped as positives and were observed in 72 out of 89 (80.9%) invasive breast tumours (Table 2). In contrast, nearly all of normal breast tissues showed only basal or low level of staining (scored 0 and 1) under the same staining condition (Table 2). Interestingly, increased PDK-1 phosphorylayion was significantly correlated with invasive breast carcinoma (*P*< 0.05) as illustrated by the Student's *t*-test using SPSS software (Table 2). Importantly, similar results were obtained using Western blottings demonstrating that PDK-1 phosphorylayion levels were remarkably elevated in MCF-7 and MDA-MB-468 breast cancer cell lines compared to HMEC and MCF-10A cells (Figure 3A). Taken together, our data thus suggest PDK-1 might play an important role in pathogenesis of breast carcinomas. Moreover, phosphorylated PDK-1 was predominately cytoplasmic localised (Figure 1), which was in close agreement with other finding suggested that retention of PDK-1 in cytoplasm provides close proximity in signalling its downstream targets in breast carcinomas (Lim *et al*, 2003). Furthermore, the expression of total PDK-1 (both phosphorylated and unphosphorylated) negligibly differed among all TMA tissues, indicating the differences in the degree and frequency of phosphorylation was more likely resulted from differential phosphorylation levels rather than quantitative variations (top row of Figure 1).

### Phosphorylation of AKT in breast carcinoma

**Figure 2:**
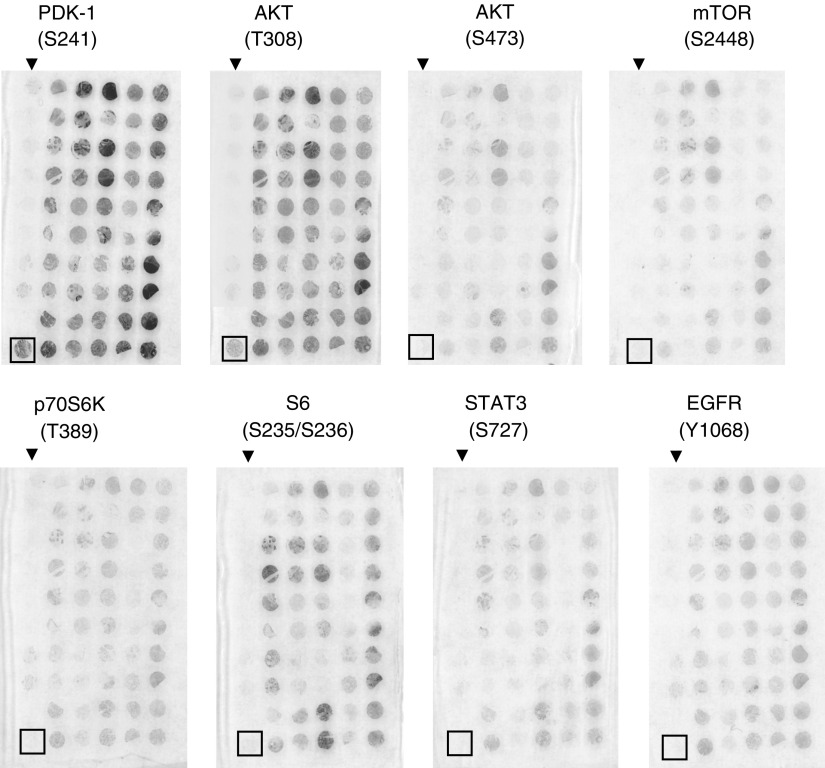
Gross view of IHC staining on TMA slides fixed with normal breast tissues and invasive breast tumours. Representative examples of IHC stained TMA slides to detect phosphorylated molecules as described in the legend to Figure 1. Nuclear staining with haematoxylin was omitted and hence red outcome indicates phosphorylated proteins in breast tissues. Noticeably, the normal breast tissues, arrayed in the left-hand row and labeled with a black arrow, revealed poor phosphorylation and remained colourless upon IHC staining. The most bottom spot (black square) of the normal tissue row was derived from placenta, which served as examples of positive staining for phosphorylation of PDK-1 (S241) and AKT (T308). The spot right above the placenta tissue was a paraffin control, which contained no tissue, whereas the remaining spots contained normal breast tissue in duplicates.

The oncogenic effects of AKT in human cancers depended on its ability of inducing multiple downstream cascades to promote cell survival, tumour growth and progression (Bellacosa *et al*, 1998; Franke *et al*, 2003; Osaki *et al*, 2004). Deregulation of this pathway leads to upregulation of AKT signalling cascades was commonly found in variety of human cancers including breast carcinoma (Vivanco and Sawyers, 2002; Lee *et al*, 2005; Levine *et al*, 2005). It was evidenced that AKT signalling was transduced from upstream PI3-K (Franke *et al*, 1995). However, the molecular linkage between PI3-K and AKT in the primary breast tumours remained to be illustrated. Although *in vitro* model demonstrated that PDK-1 is the AKT (T308) kinase (Alessi *et al*, 1997; Wick *et al*, 2000b; Williams *et al*, 2000), the clinical association between these two molecules remained to be validated. In this study, we have demonstrated significant correlation in these two kinases regarding their phosphorylation profiles in primary breast tumours. Similar to the increased phosphorylation frequency observed in PDK-1(S241), moderate to high levels of AKT(T308) phosphorylation was observed in 72 out of 89 (80.9%) of invasive breast tumours (Table 2, Figure 2). In addition, the cellular localisation of AKT(T308) closely resembled that of PDK-1, altogether suggested both molecules were correlated *in vivo* and they may involve in the same signalling cascade in primary breast tumour.

In addition to T308, S473 is another phosphorylation residue on AKT that was targeted by different kinase(s) (Hresko *et al*, 2003; Feng *et al*, 2004; Partovian and Simons, 2004; Sarbassov *et al*, 2005). In this study, however, the frequency of AKT(S473) phosphorylation in breast cancer was lower (34.8%) than those of AKT (T308) and PDK-1(S241) (Tables 2 and 3, Figure 2). Interestingly, all (100%) tumours that experienced elevated phosphorylation on AKT (S473) (scores 2 or greater) also revealed increased phosphorylation on AKT(T308) (data not shown), indicating AKT(S473) very likely prelude by phosphorylation of AKT(T308). Moreover, AKT(S473) localised in both cytoplasmic and cell membrane whereas phosphorylated AKT (T308) was predominant in the cytoplasm (Figure 1). Nevertheless, phosphorylation of AKT on both residues, T308 and S473, was significantly associated with invasive breast tumours (*P*<0.05) as shown in Table 2.

**Figure 3:**
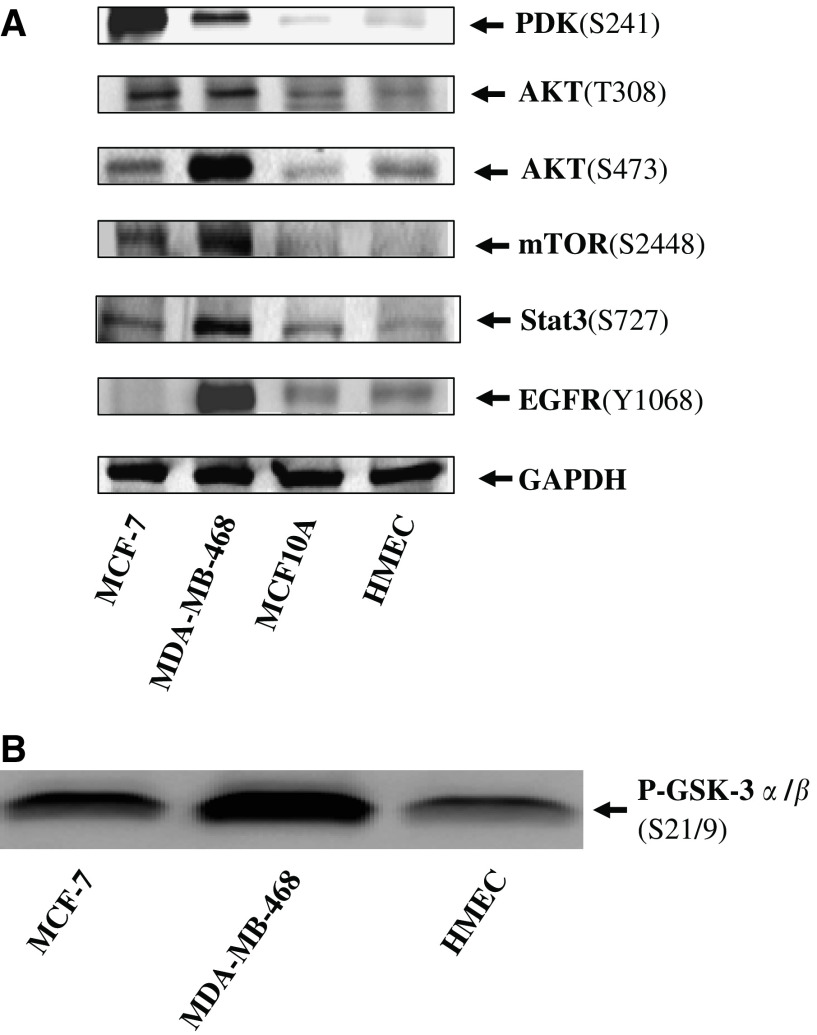
Elevated protein phosphorylation and AKT kinase activity in human breast cancer lines. Subconfluent proliferating culture of breast cancer lines (MCF-7 and MDA-MB-468), immortalised breast epithelial line (MCF10A), and normal primary HMEC were harvested and lysed. Proteins (100 *μ*g) derived from such lysate were subjected to Western blot analysis using phosphor-specific primary antibodies to detect various phosphorylated proteins (**A**). In addition, 500 *μ*g of protein from the same lysates were immunoprecipitated with anti-AKT antibody and the resulting immune complexes were further subjected to AKT kinase activity assays using GSK-3*α*/*β* as a substrate. The presence of phosphorylated GSK-3*α*/*β* (Ser21/Ser9) indicates positive AKT kinase activity in cells (**B**).

To validate our observations further, two independent studies were carried out to reveal the phosphorylation status and kinase activation of AKT in breast cancer cell lines. As shown in Figure 3A, AKT phosphorylation at T308 was significantly higher (and nearly equally) in both cancer lines than in HMEC and in non-cancerous MCF10A cells. However, phosphorylation at AKT(S473) was variable between two cancer lines. As shown in Figure 3A, MDA-MB-468 harboured remarkable elevation whereas MCF-7 contained modest increase (Figure 3A). In addition, MDA-MB-468 also retained far more AKT kinase activity than MCF-7, although they both had higher AKT kinase activity than HMEC (Figure 3B). The profile of AKT kinase activities in normal and cancer cells resembled more close to S473 than T308 (Figure 3A and B), which is again similar to the finding from Kinexus kinase assay on S473 (Table 1). Taken together, our data suggest that activation of AKT pathway is consistently higher in breast cancer lines than in noncancerous breast epithelia, and hence suggest that AKT activation may play critical roles in the tumorigenesis of human mammary cancer.

### Elevated phosphorylation of mTOR/p70S6K/S6 pathway in breast cancer

AKT promotes oncogenesis through phosphorylation of several downstream targets. Among them, mTOR(S2448) was phosphorylated directly by AKT following growth factor stimulation (Reynolds *et al*, 2002). Phosphorylation of mTOR(S2448) has been suggested to be an important hallmark for such activated pathway, which subsequently signals downstream targets including p70S6K and S6 (Martin and Blenis, 2002; Yonezawa *et al*, 2004). We have found 40 (44.9%) breast cancer tissues revealed moderate to high levels of mTOR(S2448) phosphorylation, whereas 64 (71.9%) and 52 (58.5%) out of 89 invasive breast carcinoma gave rise to positive staining (scores 2 and 3) on phosphorylated p70S6K(T389) and S6(S235/236), respectively (Table 2). Nevertheless, phosphorylation of all three molecules were positively correlated with invasive breast carcinoma (*P*<0.05) (Table 2) in this study. Interestingly, the cellular localisations among these three kinases were strikingly different. Phosphorylated mTOR and S6 resided predominately in the cytoplasm whereas phosphorylated p70S6K were predominately in the nuclei, suggesting trafficking mechanisms were involved to transduce signals between cytoplasm and nucleus compartments.

Moreover, the phosphorylation status of mTOR(S2448) was further examined in breast cancer lines and in HMEC using Western blotting. As demonstrated in Figure 3A, dramatic elevated phosphorylation of mTOR(S2448) was consistently observed in both breast cancer lines, but was nearly undetectable in primary HMEC or in MCF10A. It thus implicates that maintaining mTOR in highly phosphorylated and activated state may attribute breast cancer.

### Phosphorylation of Stat3 in breast cancer

Numerous studies suggested constitutively activated Stat3 plays an oncogenic role in many types in human cancers including breast cancer (Bowman *et al*, 2000; Garcia *et al*, 2001). Activation of Stat3 requires phosphorylation on two residues S727 and Y705 (Wen *et al*, 1995; Jain *et al*, 1999; Lim and Cao, 2001). mTOR is one of the key protein kinases that phosphorylates Stat3 at S727 (Calo *et al*, 2003; Clevenger, 2004; Yonezawa *et al*, 2004). We thereby examined Stat3 phosphorylation on S727 using Western blotting. As shown in Figure 3A, elevated phosphorylation of Stat3(S727) was prominent in MDA-MB-468, modest in MCF-7, as compared to MCF10A and HMEC. It is in close agreement with data from Kinexus phosphorylated protein screening assays demonstrating phosphorylation of Stat3 at S727 was only elevated in MDA-MB-468, but not in MCF-7. All together, these results hence suggest phosphorylation of Stat3(S727) may be prevalent to higher degree of breast cancer lines such as MDA-MB-468.

Additional studies on Stat3(S727) in primary breast tumours using Stat3(Y705) as a control were carried out by applying IHC staining on TMA slides. As shown in Table 2, we found 40 (44.9%) and 23 (25.8%) out of 89 breast cancer tissues retained elevated phosphorylation on residues S727 and on Y705, respectively. Nevertheless, phosphorylated Stat3 on either residue significantly associated with invasive breast tumours (*P*<0.05, Table 2) and predominantly localised in the nuclei (Figure 1).

### Phosphorylated EGFR(Y1068) was exclusively in the nucleus of breast tumours

Although EGFR was suggested to be one of upstream regulators of PDK-1/AKT and Stat3 pathways (Engelman *et al*, 2005; Ivanov and Hei, 2005; Phillips *et al*, 2005), the role of EGFR activation plays in relation to PDK-1/AKT/mTOR/p70S6K/S6 cascade in breast cancers has not been firmly established. In this study, we have shown that moderate to high levels of EGFR phosphorylation (Y1086) were observed in 67 out of 89 (75.3%) primary breast tumours and was associated with invasive breast tumours (*P*<0.05) (Table 1). Interestingly, the phosphorylated EGFR was predominantly in the nuclei (Figure 1), which was in strong agreement as other studies, suggested that the nuclear EGFR positively associated with poor survival outcomes in patients with breast cancer (Lo *et al*, 2005). Moreover, elevated phosphorylation on EGFR(Y1068) was dramatically increased in MDA-MB-468, but not in MCF-7, as evidenced by the results from Kinexus screening assays (Table 1) and from Western blots (Figure 3A). It suggest EGFR(Y1068) might serve as a promising molecular diagnostic marker to differentiate higher stage from lower degree of breast cancer and noncancerous mammary epithelial cells.

### Concomitant phosphorylation of PDK-1 with other downstream kinases

As PDK-1 phosphorylation was implicated as an apex pivotal intracellular signalling molecules, we then investigated if phosphorylated PDK-1 positively correlates the phosphorylation of putative downstream targets in breast caricinomas. Thus, the Bivariate Pearson *χ*^2^ tests were performed to statistically relate the phosphorylation of PDK-1 to other kinases that were studied in this report. As shown in Table 2, phosphorylation of EGFR and the remaining kinases were all correlated with PDK-1 phosphorylation (*P*<0.05), suggesting that EGFR might be one of the upstream regulators whereas other remaining kinases are downstream of PDK-1 pathway in breast carcinoma. However, the correlation in the transcription factor Stat3 phosphorylation is somewhat different. Although Stat3 (S727) remains correlate well with kinases in this cascade including EGFR, PDK-1, AKT, mTOR, p70S6K and S6 (*P*<0.05 in Table 1), phosphorylated Stat3 (Y705) did not correlate with PDK-1 phosphorylation in this study (*P*>0.05; Table 2), despite it is associated with invasive breast carcinoma (*P*<0.05; Table 2). It hence suggests that Stat3 phosphorylation (Y705) was likely accomplished by different kinase(s) that were excluded from PDK-1/mTOR/p70S6K/S6K pathway and further support the notion indicating phosphorylation on these two residues was mediated by independent signalling events (Jain *et al*, 1999; Yokogami *et al*, 2000; Garcia *et al*, 2001; Lim and Cao, 2001; Yonezawa *et al*, 2004).

### Frequent elevation of PDK-1 phosphorylation at residue S241 in high stages of breast cancer

Out of total 89 breast tumours, 83 were pathologically categorised into four stages as shown in Table 3. Although the correlation between kinase phosphorylation and the stages of breast tumours was attempted, the statistically significant correlation was unachievable to be established due to low numbers at stages 1 and 3 (*N*=3 and 7, respectively). However, remarkably high frequency of moderate to high level (scores 2 and 3 in IHC staining) of phosphorylation on PDK-1(S241), AKT(T308) and p70S6K(T389) was found at high stages of breast cancer (stages 3 and 4). Up to 86% of metastasised breast tumour (stage 4) retained elevated phosphorylation on both PDK-1(S241) and AKT(308) whereas 100% of stage 3 and 76% of stage 4 revealed moderate to high level of phosphorylation on p70S6K(T389) (Table 3).

## DISCUSSION

To examine the molecular pathogenesis involving oncogene activation in breast carcinoma, we compared phosphorylation levels of 10 protein kinases as well Stat3 and c-Jun between normal primary HMEC and breast cancer cell lines and have identified the activation of PDK-1/AKT may play important roles in breast tumorigenesis. The diverse phosphorylation profiles in breast cancer lines (Table 1) suggested these molecules may attribute somewhat differently in breast tumorigenesis. Elevated phosphorylation of PDK-1, mTOR and increased AKT kinase activity in both MDA-MB-468 and MCF-7 breast cancer cell lines (Table 1, Figure 3A and B) indicated that they may play important roles in breast carcinogenesis. However, these results might be misleading because additional genetic alteration(s) or kinase activation might be generated from the long-term *in vitro* culture of these two breast cancer cell lines. We hence performed similar studies on TMA slides fixed with primary breast tumours using phosphor-specific antibodies in IHC staining assays. In our both studies, however, similar findings were obtained regardless the source of breast cancer cells (Tables 1 and 2) and thus confirmed the importance of PDK-1/AKT/mTOR/p70S6K activation in breast cancer.

Noticeably, all kinases that we examined in this study gave rise to enhanced phosphorylation in one or both breast cancer lines, with an exception of c-Jun (Table 1). In fact, the roles of c-Jun in human cancer remained controversial. Conflict reports have indicated it may function as a proapoptotic (Cuadrado *et al*, 2004; Qi *et al*, 2004) or antiapoptotic mediators (Johnsto *et al*, 1999; Huang *et al*, 2004) determined upon cellular context. However, our finding of decreased c-Jun phosphorylation in both breast cancer cell lines hence suggested that c-*Jun* was unlikely an antiapoptotic or proto-oncogene in those two breast cancer cell lines.

The pivotal roles involving activation of PDK-1 were investigated in this study. Although PDK-1 is an upstream kinase of AKT and transfection of PDK-1 cDNA can induce transformation of cells (Zeng *et al*, 2002), the activation of PDK-1 has not been demonstrated in primary breast carcinoma, yet. First, we showed that the phosphorylation PDK-1 at S241 was elevated in both human breast cancer cell lines compared to normal or immortalised mammary epithelial cell HMEC (Table 1, Figure 3A). Then similar findings were obtained as elevated phosphorylation of PDK-1 was found in 81% of invasive primary breast tumours (Table 2). This was the first report illustrating PDK-1 phosphorylation was frequently elevated and was significantly associated with the invasiveness of breast carcinoma (*P*<0.05, Table 2). Moreover, remarkably high (up to 86%) of high grades and metastasised breast tumours retained moderate to high level of phosphorylation on PDK-1(S241) (Table 3), indicating the aggressive metastasis of breast cancers somehow rely on the phosphorylation and subsequent activation on PDK-1.

Concomitant phosphorylation of putative downstream targets of PDK-1 was evidenced by applying IHC staining on invasive breast tumours using phospho-specific antibodies (Table 2, Figures 1 and 2). One of the most extensively studied targets of PDK-1 was AKT, which can be activated by phosphorylation on two residues (T308 and S473) for full oncogenic activity (Bellacosa *et al*, 1998). Although phosphorylation of AKT at T308 was mediated by PDK-1 (Alessi *et al*, 1997; Wick *et al*, 2000a), phosphorylation of AKT at S473 may be involved by other kinases including putative PDK-2 (Hresko *et al*, 2003), integrin-linked kinase (ILK) (Persad *et al*, 2001), protein kinase C-*α* (Partovian and Simons, 2004), DNA-dependent protein kinase C (Feng *et al*, 2004) or Rictor-mTOR complex (Sarbassov *et al*, 2005). Interestingly, we have shown the phosphorylation of AKT on T308 resembled well with that of PDK-1 in breast cancer (Tables 2 and 3, Figures 1 and 2), which agreed with other results demonstrating residue T308 of AKT was directly phosphorylated by PDK-1 (Alessi *et al*, 1997; Wick *et al*, 2000b; Williams *et al*, 2000). This is the first report that illustrated significant correlation between phosphorylation of PDK-1 and AKT (Thr308) and supported that PDK-1 is an AKT (T308) kinase in invasive breast tumours *in vivo*.

On the other hand, phosphorylation of AKT on S473 is less frequent (34.8%) and weaker in intensity in the invasive breast carcinomas (Tables 2 and 3, Figure 2) than AKT on T308 (80.9%) or PDK-1 (80.9%), suggesting that phosphorylation of former residues was less frequently detected than later ones in the invasive breast tumours. Interestingly, all (100%) of breast cancers that expressed moderate to high level of AKT(S473) concomitantly retained elevated phosphorylation on AKT(T308), indicating an *in vivo* clinical correlation between them. Perhaps, phosphorylation of AKT(T308) is a upstream prerequisite for the downstream phosphorylation on AKT(S473). This hypothesis is currently under further investigation in our laboratory.

It was reported that nuclear localisation of activated PDK-1 was to sequestrate downstream targets. As mutations that abolished cytoplasmic trafficking diminished anchorage-dependent growth and failed to protect against UV-induced apoptosis, indicating cytoplasmic localisation of PDK-1 plays pivotal roles in signalling (Lim *et al*, 2003). In our study, phosphorylated PDK-1 appears to be predominantly cytoplasmic localised as shown in Figure 1, which is the same subpopulation that activate downstream signalling cascade (Lim *et al*, 2003; Scheid *et al*, 2005). In addition, we and others have reported that phosphorylated PDK-1, AKT, mTOR, and S6K accumulate predominately in the cytoplasm, whereas EGFR, p70S6K, and Stat3 are in the nucleus. It hence suggested translocation mechanisms were involved in transmitting signals between cytoplasmic and nuclear compartments. Although the molecular mechanisms pertaining nuclear localisation of EGFR have not been identified yet, Spinophilin was reported to be one of the scaffolding molecule that binds to p70S6K (Buchsbaum *et al*, 2003).

The finding of concomitant phosphorylation of kinases downstream from PDK-1 was manifested in this study. The phosphorylation of the putative downstream kinases was positively associated with PDK-1 phosphorylation and together correlated with the invasive breast tumours (*P*<0.05, Table 2). Although we have attempted to correlate the stages and distal metastasis of breast tumours to the phosphorylation of kinases in the PDK-1/AKT pathways, we were unable to obtain statistically significant data due to insufficient numbers of breast tumours in various stages (Table 3). However, the elevated phosphorylation on mTOR and Stat3 was noticeably differed from that on PDK-1 in various stages of breast cancer, indicating these molecules are not in a single linear signalling cascade regulated by PDK-1. Instead, additional regulators (or pathways) are involved and multiple networking signalling pathways may interplay, which all together in part lead to breast tumours. For example, recent studies have suggested that TSCI and II function together to negatively regulate the insulin signalling pathways, including mTOR (Gao and Pan, 2001). Similarly, additional regulatory pathways other than PDK-1 are likely controlling Stat3 activation as demonstrated by phosphorylation on Y705 was only moderately associated with S727 and was statistically unrelated to PDK-1 phosphorylation (*P*>0.05 in Table 2). Nevertheless, we are currently investigating the molecular aetiologic mechanisms of kinase phosphorylation/activation, which perhaps involve genetic alterations leading to constitutive activation of the upstream apex receptor kinases.

In general, *in vitro* findings are preliminary in nature and require further validation *in vivo* with clinical studies or with laboratory xenograft animal models. However, our observation as frequent deregulation in the PDK-1/AKT/mTOR/p70S6K signalling pathway and its prognostic role in breast cancers implicated the notion of using inhibitors to impair this pathway and to provide additional strategy to treat breast cancer. Moreover, the detection of phosphorylated PDK-1, AKT, or mTOR through the simplicity and reproducibility of IHC staining might provide the treatment regimen that simultaneously inhibit multiple kinases within the same signalling pathway, ensure the blockage of the pathway, which ultimately reverse the malignant phenotypes. Among the options of drugs, inhibitors that abrogate the most upstream apex signalling pathways, such as PDK-1, should provide better clinical treatment efficacy as they disable far more downstream signalling molecules.

## Figures and Tables

**Table 1:** Protein phosphorylation profiles in TERT and in breast cancer lines relative to that in normal primary HMEC^a^

**Protein**	**TERT**	**MCF-7**	**MDA-MB-468**
AKT (S473)	0.43	0.96	2.87
C-Jun (S73)	0.15	0.45	0.02
C-MetR (Y1230)	1.37	0.50	1.37
EGFR (Y1068)	0.51	0.21	5.22
ErbB2/Her2 (Y1248)	0.38	0.27	4.51
FAK (Y576)	0.72	1.59	4.14
mTOR (S2448)	0.71	2.00	2.17
P70S6K (T389)	0.79	35.26	10.22
PDGFR (Y716)	0.52	0.05	8.89
PDK-1 (S241)	2.03	6.45	5.07
Shc (Y239)	1.82	0.20	3.04
Stat3 (S727)	0.26	0.77	2.71

a Phosphorylation of 10 kinases and two transcription factors (c-Jun and Stat3) in TERT and in breast cancer cell lines (MCF-7 and MDA-MBB-468) relative to that in normal primary HEMC, which was set as value 1.0. Data were derived from the protein phosphorylation analysis using Kinexus KCPS-1.0 phosphoprotein profiling screen software as described in the Materials and methods.

**Table 2:** Elevated protein phosphorylation correlated with invasion of breast cancer and with phosphorylation of PDK-1

	**Number of positives**			
	**Normal**	**Cancer**	***P-*value**	
**Protein phosphorylation^a^**	***N*=6^a^**	**89^a^**	**Invasion^b^**	**PDK-1 phosphorylation^c^**
PDK-1 (S241)	0 (0%)	72 (80.9%)	<0.05	
AKT (T308)	0 (0%)	72 (80.9%)	<0.05	<0.05
AKT (S473)	0 (0%)	31 (34.8%)	<0.05	<0.05
mTOR (S2448)	0 (0%)	40 (44.9%)	<0.05	<0.05
P70S6K (T389)	0 (0%)	64 (71.9%)	<0.05	<0.05
S6 (S235/236)	0 (0%)	52 (58.5%)	<0.05	<0.05
Stat3 (S727)	0 (0%)	40 (44.9%)	<0.05	<0.05
Stat3 (Y705)	0 (0%)	23 (25.8%)	<0.05	>0.05
EGFR (Y1068)	0 (0%)	67 (75.3%)	<0.05	<0.05

a The total numbers of breast tissues on TMA slides that experienced positive outcomes (scores 2 and 3) following IHC staining using various phospho-specific antibodies (as described in the Materials and methods) were counted. The resulting numbers were divided by either 6 (total number of normal breast tissue) or 89 (total number of invasive breast carcinoma) to obtain percentage values shown in the parenthesis.

b Student's *t*-test was to analyse whether phosphorylated kinases correlated with invasive breast carcinomas.

c Bivariate Pearson *χ*^2^ test was to correlate the phosphorylation of PDK-1 to the remaining kinases. Both statistic analyses were carried out using SPSS software version 13.0 (SPSS Inc., Chicago, IL, USA). Significant correlation is rendered if the correlation coefficient *P* is less than 0.05.

**Table 3:** Frequency of elevated protein phosphorylation at various stages of breast cancer

	**Number of positives**			
**Stages**	**I**	**II**	**III**	**IV**
**Phosphorylated protein^a^**	***N*=3**	**52**	**7**	**21**
PDK-1 (S241)	1 (33%)	42 (80%)	6 (86%)	18 (86%)
AKT (T308)	1 (33%)	43 (83%)	6 (86%)	18 (86%)
AKT (S473)	1 (33%)	20 (39%)	1 (14%)	6 (29%)
mTOR (S2448)	1 (33%)	25 (48%)	2 (29%)	8 (38%)
P70S6K (T389)	1 (33%)	36 (69%)	7 (100%)	16 (76%)
S6 (S235/236)	2 (66%)	29 (56%)	4 (57%)	14 (67%)
Stat3 (S727)	1 (33%)	27 (52%)	1 (14%)	7 (33%)
EGFR (Y1068)	2 (66%)	42 (81%)	3 (43%)	14 (67%)

a Number of breast tumours in each stage that contained positive protein phosphorylation (scores 2 and 3 upon IHC staining on TMA slides) was counted and was divided to the total number (*N*) of breast tumours in each group, which gave rise to the frequency (%) of tumours that retained elevated protein phosphorylation. The tumours were classified according to the TNM systems (Elston and Ellis, 1991) (T: invasion of primary tumour; N: metastasis to regional lymph nodes; M: metastasis to distal organs) as well as the guidelines from American Cancer Society.

## References

[bib1] Alessi D, James S, Downes C, Holmes A, Gaffney P, Reese C, Cohen P (1997) Characterization of a 3-phosphoinositide-dependent protein kinase which phosphorylates and activates protein kinase Balph. Curr Biol 7: 261–269 9094314 10.1016/s0960-9822(06)00122-9

[bib2] Balendran A, Biondi R, Cheung P, Casamayor A, Deak M, Alessi D (2000) A 3-phosphoinositide-dependent protein kinase-1 (PDK1) docking site is required for the phosphorylation of protein kinase Czeta (PKCzeta) and PKC-related kinase 2 by PDK1. J Biol Chem 275: 20806–20813 10764742 10.1074/jbc.M000421200

[bib3] Bellacosa A, Chan T, Ahmed N, Datta K, Malstrom S, Stokoe D, McCormick F, Feng J, Tsichlis P (1998) Akt activation by growth factors is a multiple-step process: the role of the PH domain. Oncogene 17: 313–325 9690513 10.1038/sj.onc.1201947

[bib4] Bowman T, Garcia R, Turkson J, Jove R (2000) STATs in oncogenesis. Oncogene 19: 2474–2488 10851046 10.1038/sj.onc.1203527

[bib5] Buchsbaum R, Connolly B, Feig L (2003) Regulation of p70 S6 kinase by complex formation between the Rac guanine nucleotide exchange factor (Rac-GEF) Tiam1 and the scaffold spinophilin. J Biol Chem 278: 18833–18841 12531897 10.1074/jbc.M207876200

[bib6] Calo V, Migliavacca M, Bazan V, Macaluso M, Buscemi M, Gebbia N, Russo A (2003) STAT proteins: from normal control of cellular events to tumorigenesis. J Cell Physiol 179: 157–168 10.1002/jcp.10364 14502555

[bib7] Carraway H, Hidalgo M (2004) New targets for therapy in breast cancer: mammalian target of rapamycin (mTOR) antagonists. Breast Cancer Res 6: 219–224 15318929 10.1186/bcr927 PMC549184

[bib8] Casamayor A, Morrice N, Alessi D (1999) Phosphorylation of Ser-241 is essential for the activity of 3-phosphoinositide-dependent protein kinase-1: identification of five sites of phosphorylation *in vivo*. Biochem J 342: 287–292 10455013 PMC1220463

[bib9] Castedo M, Ferri KF, Kroemer G (2002) Mammalian target of rapamycin (mTOR): pro- and anti-apoptotic. Cell Death Differ 9: 99–100 11840159 10.1038/sj.cdd.4400978

[bib10] Chan S (2004) Targeting the mammalian target of rapamycin (mTOR): a new approach to treating cancer. Br J Cancer 91: 1420–1424 15365568 10.1038/sj.bjc.6602162 PMC2409926

[bib11] Clevenger C (2004) Roles and regulation of stat family transcription factors in human breast cancer. Am J Pathol 165: 1449–1460 15509516 10.1016/S0002-9440(10)63403-7 PMC1618660

[bib12] Cuadrado A, Gonzalez L, Suarez Y, Martinez T, Munoz A (2004) JNK activation is critical for Aplidin-induced apoptosis. Oncogene 23: 4673–4680 15122339 10.1038/sj.onc.1207636

[bib13] Dankort D, Maslikowski B, Warner N, Kanno N, Kim H, Wang Z, Moran M, Oshima R, Cardiff R, Muller W (2001) Grb2 and Shc adapter proteins play distinct roles in Neu (ErbB-2)-induced mammary tumorigenesis: implications for human breast cancer. Mol Cell Biol 21(5): 1540–1551 11238891 10.1128/MCB.21.5.1540-1551.2001 PMC86700

[bib14] Darnell JJ, Kerr I, Stark G (1994) Jak-STAT pathways and transcriptional activation in response to IFNs and other extracellular signaling proteins. Science 264: 1415–1421 8197455 10.1126/science.8197455

[bib15] Del Peso L, Gonzalez-Garcia M, Page C, Herrera R, Nunez G (1997) Interleukin-3-induced phosphorylation of BAD through the protein kinase Akt. Science 278: 687–689 9381178 10.1126/science.278.5338.687

[bib16] Edakuni G, Sasatomi E, Satoh T, Tokunaga O, Miyazaki K (2001) Expression of the hepatocyte growth factor/c-Met pathway is increased at the cancer front in breast carcinoma. Pathol Int 51: 172–178 11328532 10.1046/j.1440-1827.2001.01182.x

[bib17] Elliott B, Hung W, Boag A, Tuck A (2002) The role of hepatocyte growth factor (scatter factor) in epithelial–mesenchymal transition and breast cancer. Can J Physiol Pharmacol 80: 91–102 11934261 10.1139/y02-010

[bib18] Elston CW, Ellis IO (1991) Pathological prognostic factors in breast cancer. I. The value of histological grade in breast cancer: evidence from a large study with long-term follow-up. Histopathology 19: 403–410 1757079 10.1111/j.1365-2559.1991.tb00229.x

[bib19] Engelman JA, Jänne PA, Mermel C, Pearlberg J, Mukohara T, Fleet C, Cichowski K, Johnson BE, Cantley LC (2005) ErbB-3 mediates phosphoinositide 3-kinase activity in gefitinib-sensitive non-small cell lung cancer cell lines. Proc Natl Acad Sci USA 102: 3788–3793 15731348 10.1073/pnas.0409773102 PMC553328

[bib20] Feng J, Park J, Cron P, Hess D, Hemmings B (2004) Identification of a PKB/Akt hydrophobic motif Ser-473 kinase as DNA-dependent protein kinase. J Biol Chem 279: 41189–41196 15262962 10.1074/jbc.M406731200

[bib21] Finlayson C, Chappell J, Leitner J, Goalstone M, Garrity M, Nawaz S, Ciaraldi T, Draznin B (2003) Enhanced insulin signaling via Shc in human breast cancer. Metabolism 52: 1606–1611 14669164 10.1016/s0026-0495(03)00311-1

[bib22] Franke T, Hornik C, Segev L, Shostak G, Sugimoto C (2003) PI3K/Akt and apoptosis: size matters. Oncogene 22: 8983–8998 14663477 10.1038/sj.onc.1207115

[bib23] Franke T, Kaplan D, Cantley L (1997) PI3K: downstream AKTion blocks apoptosis. Cell 88: 435–437 9038334 10.1016/s0092-8674(00)81883-8

[bib24] Franke T, Yang S, Chan T, Datta K, Kazlauskas A, Morrison D, Kaplan D, Tsichlis P (1995) The protein kinase encoded by the Akt proto-oncogene is a target of the PDGF-activated phosphatidylinositol 3-kinase. Cell 81: 727–736 7774014 10.1016/0092-8674(95)90534-0

[bib25] Fu A, Fu W, Ng A, Chien W, Ng Y, Wang J, Ip N (2004) Cyclin-dependent kinase 5 phosphorylates signal transducer and activator of transcription 3 and regulates its transcriptional activity. Proc Natl Acad Sci USA 101: 6728–6733 15096606 10.1073/pnas.0307606100 PMC404113

[bib26] Gao X, Pan D (2001) TSC1 and TSC2 tumor suppressors antagonize insulin signaling in cell growth. Genes Dev 15: 1383–1392 11390358 10.1101/gad.901101 PMC312704

[bib27] Garcia R, Bowman T, Niu G, Yu H, Minton S, Muro-Cacho C, Cox C, Falcone R, Fairclough R, Parsons S, Laudano A, Gazit A, Levitzki A, Kraker A, Jove R (2001) Constitutive activation of Stat3 by the Src and JAK tyrosine kinases participates in growth regulation of human breast carcinoma cells. Oncogene 20: 2499–2513 11420660 10.1038/sj.onc.1204349

[bib28] Gingras A, Gygi S, Raught B, Polakiewicz R, Abraham R, Hoekstra M, Aebersold R, Sonenberg N (1999) Regulation of 4E-BP1 phosphorylation: a novel two-step mechanism. Genes Dev 13: 1422–1437 10364159 10.1101/gad.13.11.1422 PMC316780

[bib29] Hresko R, Murata H, Mueckler M (2003) Phosphoinositide-dependent kinase-2 is a distinct protein kinase enriched in a novel cytoskeletal fraction associated with adipocyte plasma membranes. J Biol Chem 278: 21615–21622 12682057 10.1074/jbc.M302937200

[bib30] Huang Y, Keen JC, Hager E, Smith R, Hacker A, Frydman B, Valasinas AL, Reddy VK, Marton LJ, Casero RAJ, Davidson NE (2004) Regulation of polyamine analogue cytotoxicity by c-Jun in human MDA-MB-435 cancer cells. Mol Cancer Res 2: 81–88 14985464

[bib31] Ivanov VN, Hei TK (2005) Combined treatment with EGFR inhibitors and arsenite upregulated apoptosis in human EGFR-positive melanomas: a role of suppression of the PI3K-AKT pathway. Oncogene 24: 616–626 15580309 10.1038/sj.onc.1208125 PMC4394621

[bib32] Jain N, Zhang T, Kee W, Li W, Cao X (1999) Protein kinase C delta associates with and phosphorylates Stat3 in an interleukin-6-dependent manner. J Biol Chem 274: 24392–24400 10446219 10.1074/jbc.274.34.24392

[bib33] Johnsto SR, Lu B, Scott GK, Kushner PJ, Smith IE, Dowsett M, Benz CC (1999) Increased activator protein-1 DNA binding and c-Jun NH2-terminal kinase activity in human breast tumors with acquired tamoxifen resistance. Clin Cancer Res 5: 251–256 10037172

[bib34] Krueger JS, Keshamouni VG, Atanaskova N, Reddy KB (2001) Temporal and quantitative regulation of mitogen-activated protein kinase (MAPK) modulates cell motility and invasion. Oncogene 20: 4209–4218 11464287 10.1038/sj.onc.1204541

[bib35] Kulik G, Klippel A, Weber MJ (1997) Antiapoptotic signalling by the insulin-like growth factor I receptor, phosphatidylinositol 3-kinase, and Akt. Mol Cell Biol 17: 1595–1606 9032287 10.1128/mcb.17.3.1595 PMC231885

[bib36] Kusaba H, Ghosh P, Derin R, Buchholz M, Sasaki C, Madara K, Longo D (2004) Interleukin-12-induced IFN-gamma production by human peripheral blood T cells is regulated by mammalian target of rapamycin (mTOR). J Biol Chem 280: 1037–1043 15522880 10.1074/jbc.M405204200

[bib37] Le Good J, Ziegler W, Parekh D, Alessi D, Cohen P, Parker P (1998) Protein kinase C isotypes controlled by phosphoinositide 3-kinase through the protein kinase PDK1. Science 281: 2042–2045 9748166 10.1126/science.281.5385.2042

[bib38] Lee JW, Soung YH, Kim SY, Lee HW, Park WS, Nam SW, Kim SH, Lee JY, Yoo NJ, Lee SH (2005) PIK3CA gene is frequently mutated in breast carcinomas and hepatocellular carcinomas. Oncogene 24: 1477–1480 15608678 10.1038/sj.onc.1208304

[bib39] Lemmon M (2003) The EGF receptor family as therapeutic targets in breast cancer. Breast Dis 18: 33–43 15687687 10.3233/bd-2003-18105

[bib40] Lengyel E, Prechtel D, Resau J, Gauger K, Welk A, Lindemann K, Salanti G, Richter T, Knudsen B, Vande Woude G, Harbeck N (2005) C-Met overexpression in node-positive breast cancer identifies patients with poor clinical outcome independent of Her2/neu. Int J Cancer 4: 678–682 10.1002/ijc.20598 15455388

[bib41] Levine DA, Bogomolniy F, Yee CJ, Lash A, Barakat RR, Borgen PI, Boyd J (2005) Frequent mutation of the PIK3CA gene in ovarian and breast cancers. Clin Cancer Res 8: 2875–2878 10.1158/1078-0432.CCR-04-2142 15837735

[bib42] Lim C, Cao X (2001) Regulation of Stat3 activation by MEK kinase 1. J Biol Chem 276: 21004–21011 11278353 10.1074/jbc.M007592200

[bib43] Lim MA, Kikani CK, Wick MJ, Dong LQ (2003) Nuclear translocation of 3′-phosphoinositide-dependent protein kinase 1 (PDK-1): a potential regulatory mechanism for PDK-1 function. Proc Natl Acad Sci USA 100: 14006–14011 14623982 10.1073/pnas.2335486100 PMC283536

[bib44] Lo H-W, Xia W, Wei Y, Ali-Seyed M, Huang S, Hung M (2005) Novel prognostic value of nuclear epidermal growth factor receptor in breast cancer. Cancer Res 65: 338–348 15665312

[bib45] Martin K, Blenis J (2002) Coordinate regulation of translation by the PI 3-kinase and mTOR pathways. Adv Cancer Res 86: 1–39 12374276 10.1016/s0065-230x(02)86001-8

[bib46] Mauro L, Sisci D, Bartucci M, Salerno M, Kim J, Tam T, Guvakova M, Ando S, Surmacz E (1999) SHC-alpha5beta1 integrin interactions regulate breast cancer cell adhesion and motility. Exp Cell Res 252: 439–448 10527634 10.1006/excr.1999.4639

[bib47] Mora A, Komander D, van Aalten D, Alessi D (2004) PDK1, the master regulator of AGC kinase signal transduction. Semin Cell Dev Biol 15: 161–170 15209375 10.1016/j.semcdb.2003.12.022

[bib48] Nave B, Ouwens M, Withers D, Alessi D, Shepherd P (1999) Mammalian target of rapamycin is a direct target for protein kinase B: identification of a convergence point for opposing effects of insulin and amino-acid deficiency on protein translation. Biochem J 344: 427–431 10567225 PMC1220660

[bib49] Noh W, Mondesire W, Peng J, Jian W, Zhang H, Dong J, Mills G, Hung M, Meric-Bernstam F (2004) Determinants of rapamycin sensitivity in breast cancer cells. Clin Cancer Res 10: 1013–1023 14871980 10.1158/1078-0432.ccr-03-0043

[bib50] Nojima H, Tokunaga C, Eguchi S, Oshiro N, Hidayat S, Yoshino K, Hara K, Tanaka N, Avruch J, Yonezawa K (2003) The mammalian target of rapamycin (mTOR) partner, raptor, binds the mTOR substrates p70 S6 kinase and 4E-BP1 through their TOR signaling (TOS) motif. J Biol Chem 278: 15461–15464 12604610 10.1074/jbc.C200665200

[bib51] Osaki M, Oshimura M, Ito H (2004) PI3K-Akt pathway: its functions and alterations in human cancer. Apoptosis 96: 667–676 10.1023/B:APPT.0000045801.15585.dd 15505410

[bib52] Pandolfi P (2004) Breast cancer – loss of PTEN predicts resistance to treatment. N Engl J Med 351: 2337–2338 15564551 10.1056/NEJMcibr043143

[bib53] Parr C, Jiang W (2001) Expression of hepatocyte growth factor/scatter factor, its activator, inhibitors and the c-Met receptor in human cancer cells. Int J Oncol 19: 857–863 11562767

[bib54] Partovian C, Simons M (2004) Regulation of protein kinase B/Akt activity and Ser473 phosphorylation by protein kinase Calpha in endothelial cells. Cell Signal 16: 951–957 15157674 10.1016/j.cellsig.2004.01.008

[bib55] Persad S, Attwell S, Gray V, Mawji N, Deng J, Leung D, Yan J, Sanghera J, Walsh M, Dedhar S (2001) Regulation of protein kinase B/Akt-serine 473 phosphorylation by integrin-linked kinase: critical roles for kinase activity and amino acids arginine 211 and serine 343. J Biol Chem 276: 27462–27469 11313365 10.1074/jbc.M102940200

[bib56] Phillips RJ, Mestas J, Gharaee-Kermani M, Burdick MD, Sica A, Belperio JA, Keane MP, Strieter RM (2005) Epidermal growth factor and hypoxia-induced expression of CXCR4 on non-small cell lung cancer cells is regulated by the PI3-kinase/PTEN/AKT/mTOR signaling pathway and activation of HIF-1a. J Biol Chem 280: 22473–22481 15802268 10.1074/jbc.M500963200

[bib57] Pullen N, Dennis P, Andjelkovic M, Dufner A, Kozma S, Hemmings B, Thomas G (1998) Phosphorylation and activation of p70s6k by PDK1. Science 279: 707–710 9445476 10.1126/science.279.5351.707

[bib58] Qi X, Borowicz S, Pramanik R, Schultz RM, Han J, Chen G (2004) Estrogen receptor inhibits c-Jun-dependent stress-induced cell death by binding and modifying c-Jun activity in human breast cancer cells. J Biol Chem 279: 6769–6777 14638681 10.1074/jbc.M311492200

[bib59] Reynolds T, Bodine S, Lawrence JJ (2002) Control of Ser2448 phosphorylation in the mammalian target of rapamycin by insulin and skeletal muscle load. J Biol Chem 277: 17657–17662 11884412 10.1074/jbc.M201142200

[bib60] Saal L, Holm K, Maurer M, Memeo L, Su T, Wang X, Yu J, Malmstrom P, Mansukhani M, Enoksson J, Hibshoosh H, Borg A, Parsons R (2005) PIK3CA mutations correlate with hormone receptors, node metastasis, and ERBB2, and are mutually exclusive with PTEN loss in human breast carcinoma. Cancer Res 7: 2554–2559 10.1158/0008-5472-CAN-04-3913 15805248

[bib61] Sarbassov D, Guertin D, Ali S, Sabatini D (2005) Phosphorylation and regulation of Akt/PKB by the rictor–mTOR complex. Science 307: 1098–1101 15718470 10.1126/science.1106148

[bib62] Scheid MP, Parsons M, Woodgett JR (2005) Phosphoinositide-dependent phosphorylation of PDK1 regulates nuclear translocation. Mol Cell Biol 25: 2347–2363 15743829 10.1128/MCB.25.6.2347-2363.2005 PMC1061613

[bib63] Schmitz K, Grabellus F, Callies R, Otterbach F, Wohlschlaeger J, Levkau B, Kimmig R, Schmid K, Baba H (2005) High expression of focal adhesion kinase (p125FAK) in node-negative breast cancer is related to overexpression of HER-2/neu and activated Akt kinase but does not predict outcome. Breast Cancer Res 7: 194–203 10.1186/bcr977 PMC1064131 15743500

[bib64] Scott P, Brunn G, Kohn A, Roth R, Lawrence JJ (1998) Evidence of insulin-stimulated phosphorylation and activation of the mammalian target of rapamycin mediated by a protein kinase B signaling pathway. Proc Natl Acad Sci USA 95: 7772–7777 9636226 10.1073/pnas.95.13.7772 PMC22753

[bib65] Sekulic A, Hudson C, Homme J, Yin P, Otterness D, Karnitz L, Abraham R (2000) A direct linkage between the phosphoinositide 3-kinase-AKT signaling pathway and the mammalian target of rapamycin in mitogen-stimulated and transformed cells. Cancer Res 60: 3504–3513 10910062

[bib66] Shao Z, Nguyen M, Barsky S (2000) Human breast carcinoma desmoplasia is PDGF initiated. Oncogene 19(38): 4337–4345 10980609 10.1038/sj.onc.1203785

[bib67] Storz P, Toker A (2002) 3′-phosphoinositide-dependent kinase-1 (PDK-1) in PI 3-kinase signaling. Front Biosci 7: 886–902 10.2741/storz 11897568

[bib68] Toker A, Newton A (2000) Cellular signaling: pivoting around PDK-1. Cell 103: 185–188 11057891 10.1016/s0092-8674(00)00110-0

[bib69] Vivanco I, Sawyers CL (2002) The phosphatidylinositol 3-Kinase AKT pathway in human cancer. Nat Rev Cancer 2: 489–501 12094235 10.1038/nrc839

[bib70] von Manteuffel S, Dennis P, Pullen N, Gingras A, Sonenberg N, Thomas G (1997) The insulin-induced signalling pathway leading to S6 and initiation factor 4E binding protein 1 phosphorylation bifurcates at a rapamycin-sensitive point immediately upstream of p70s6k. Mol Cell Biol 17: 5426–5436 9271419 10.1128/mcb.17.9.5426 PMC232392

[bib71] Wang HY, Cheng Z, Malbon CC (2003) Overexpression of mitogen-activated protein kinase phosphatases MKP1, MKP2 in human breast cancer. Cancer Lett 191: 229–237 12618338 10.1016/s0304-3835(02)00612-2

[bib72] Wen Z, Zhong Z, Darnell JJ (1995) Maximal activation of transcription by Stat1 and Stat3 requires both tyrosine and serine phosphorylation. Cell 82: 241–250 7543024 10.1016/0092-8674(95)90311-9

[bib73] Wick M, Dong L, Riojas R, Ramos F, Liu F (2000a) Mechanism of phosphorylation of protein kinase B/Akt by a constitutively active 3-phosphoinositide-dependent protein kinase-1. J Biol Chem 275: 40400–40406 11006271 10.1074/jbc.M003937200

[bib74] Wick M, Dong L, Riojas R, Ramos F, Liu F (2000b) Mechanism of phosphorylation of protein kinase B/Akt by a constitutively active 3-phosphoinositide-dependent protein kinase-1. J Biol Chem 275: 40400–40406 11006271 10.1074/jbc.M003937200

[bib75] Williams M, Arthur J, Balendran A, van der Kaay J, Poli V, Cohen P, Alessi D (2000) The role of 3-phosphoinositide-dependent protein kinase 1 in activating AGC kinases defined in embryonic stem cells. Curr Biol 10: 439–448 10801415 10.1016/s0960-9822(00)00441-3

[bib76] Xie Z, Zeng X, Waldman T, Glazer R (2003) Transformation of mammary epithelial cells by 3-phosphoinositide-dependent protein kinase-1 activates beta-catenin and c-Myc, and down-regulates caveolin-1. Cancer Res 63: 5370–5375 14500370

[bib77] Xu G, Zhang W, Bertram P, Zheng XF, McLeod H (2004) Pharmacogenomic profiling of the PI3K/PTEN-AKT-mTOR pathway in common human tumors. Int J Oncol 4: 893–900 15010827

[bib78] Yokogami K, Wakisaka S, Avruch J, Reeves S (2000) Serine phosphorylation and maximal activation of STAT3 during CNTF signaling is mediated by the rapamycin target mTOR. Curr Biol 10: 47–50 10660304 10.1016/s0960-9822(99)00268-7

[bib79] Yonezawa K, Yoshino KI, Tokunaga C, Hara K (2004) Kinase activities associated with mTOR. Curr Top Microbiol Immunol 279: 271–282 14560963 10.1007/978-3-642-18930-2_16

[bib80] Zeng X, Xu H, Glazer R (2002) Transformation of mammary epithelial cells by 3-phosphoinositide-dependent protein kinase-1 (PDK1) is associated with the induction of protein kinase Calpha. Cancer Res 62: 3538–3543 12068001

[bib81] Zhang P, Ostrander J, Faivre E, Olsen A, Fitzsimmons D, Lange C (2005) Regulated association of protein kinase B/Akt with breast tumor kinase. J Biol Chem 280: 1982–1991 15539407 10.1074/jbc.M412038200

[bib82] Zhong Z, Wen Z, Darnell JJ (1994) Stat3: a STAT family member activated by tyrosine phosphorylation in response to epidermal growth factor and interleukin-6. Science 264: 95–98 8140422 10.1126/science.8140422

[bib83] Zhou X, Tan M, Stone HV, Klos K, Lan K, Yang Y, Yang W, Smith T, Shi D, Yu D (2004) Activation of the Akt/mammalian target of rapamycin/4E-BP1 pathway by ErbB2 overexpression predicts tumor progression in breast cancers. Clin Cancer Res 10: 6779–6788 15501954 10.1158/1078-0432.CCR-04-0112

